# Genetic variation of *TLR4 *influences immunoendocrine stress response: an observational study in cardiac surgical patients

**DOI:** 10.1186/cc10130

**Published:** 2011-04-05

**Authors:** Alexander Koch, Lutz Hamann, Matthias Schott, Olaf Boehm, Dirk Grotemeyer, Muhammed Kurt, Carsten Schwenke, Ralf R Schumann, Stefan R Bornstein, Kai Zacharowski

**Affiliations:** 1 Clinic of Anaesthesiology, Intensive Care Medicine and Pain Therapy, J.W.-Goethe-University Hospital, Theodor-Stern-Kai 7, Frankfurt am Main 60590, Germany; 2 Institute for Microbiology and Hygiene, Charite-University Medical Center Berlin, Dorotheenstrasse 96, Berlin 10117, Germany; 3 Department of Endocrinology, Diabetes, and Rheumatology, University Hospital Duesseldorf, Moorenstrasse 5, Duesseldorf 40225, Germany; 4 Department of Anesthesiology and Intensive Care, University Hospital Bonn, Sigmund-Freud-Strasse 25, Bonn 53105, Germany; 5 Service de Chirurgie Vasculaire, Centre Hospitalier du Kirchberg, 9, rue Edward Steichen, Luxembourg 2540, Luxembourg; 6 Department of Thoracic and Cardiovascular Surgery, University Hospital Duesseldorf, Moorenstrasse 5, Duesseldorf 40225, Germany; 7 SCOSSiS Statistical consulting, Zeltinger Strasse 58 G, Berlin 13465, Germany; 8 Department of Medicine III, University Hospital Carl Gustav Carus, Technische Universität Dresden, Fetscherstrasse 74, Dresden 01307, Germany

## Abstract

**Introduction:**

Systemic inflammation (for example, following surgery) involves Toll-like receptor (TLR) signaling and leads to an endocrine stress response. This study aims to investigate a possible influence of *TLR2 *and *TLR4 *single nucleotide polymorphisms (SNPs) on perioperative adrenocorticotropic hormone (ACTH) and cortisol regulation in serum of cardiac surgical patients. To investigate the link to systemic inflammation in this context, we additionally measured 10 different cytokines in the serum.

**Methods:**

A total of 338 patients admitted for elective cardiac surgery were included in this prospective observational clinical cohort study. Genomic DNA of patients was screened for *TLR2 *and *TLR4 *SNPs. Serum concentrations of ACTH, cortisol, interferon (IFN)-γ, interleukin (IL)-1β, IL-2, IL-4, IL-5, IL-6, IL-8, IL-10, tumor necrosis factor (TNF)-α and granulocyte macrophage-colony stimulating factor (GM-CSF) were determined before surgery, immediately post surgery and on the first postoperative day.

**Results:**

Thirteen patients were identified as *TLR2 *SNP carriers, 51 as *TLR4 *SNP carriers and 274 patients as non-carriers. Basal levels of ACTH, cortisol and cytokines did not differ among groups. In all three groups a significant, transient perioperative rise of cortisol could be observed. However, only in the non-carrier group this was accompanied by a significant ACTH rise. *TLR4 *SNP carriers had significant lower ACTH levels compared to non-carriers (mean (95% confidence intervals)) non-carriers: 201.9 (187.7 to 216.1) pg/ml; *TLR4 *SNP carriers: 149.9 (118.4 to 181.5) pg/ml; *TLR2 *SNP carriers: 176.4 ((110.5 to 242.3) pg/ml). Compared to non-carriers, *TLR4 *SNP carriers showed significant lower serum IL-8, IL-10 and GM-CSF peaks (mean (95% confidence intervals)): IL-8: non-carriers: 42.6 (36.7 to 48.5) pg/ml, *TLR4 *SNP carriers: 23.7 (10.7 to 36.8) pg/ml; IL-10: non-carriers: 83.8 (70.3 to 97.4) pg/ml, *TLR4 *SNP carriers: 54.2 (24.1 to 84.2) pg/ml; GM-CSF: non-carriers: 33.0 (27.8 to 38.3) pg/ml, *TLR4 *SNP carriers: 20.2 (8.6 to 31.8) pg/ml). No significant changes over time or between the groups were found for the other cytokines.

**Conclusions:**

Regulation of the immunoendocrine stress response during systemic inflammation is influenced by the presence of a *TLR4 *SNP. Cardiac surgical patients carrying this genotype showed decreased serum concentrations of ACTH, IL-8, IL-10 and GM-CSF. This finding might have impact on interpreting previous and designing future trials on diagnosing and modulating immunoendocrine dysregulation (for example, adrenal insufficiency) during systemic inflammation and sepsis.

## Introduction

Toll-like receptors (TLRs) are known to play a crucial role in the innate immune response in mammals. TLRs are involved in the recognition of pathogenic molecules like lipopolysaccharide (LPS), lipoteichoic acid (LTA), bacterial DNA and others [1]. Furthermore, there is good evidence for the involvement of TLRs in the crosstalk of immune system and the hypothalamic-pituitary-adrenal (HPA) axis [2-5]. In *TLR2 *deficient mice, adrenal glands are significantly larger compared to wild-type mice. However, the corticosterone plasma levels are significantly lower in the deficient mice. Inducing a systemic inflammation with bacterial wall components in *TLR2 *deficient mice leads to an impaired release of both corticosterone and pro-inflammatory cytokines compared to wild-type animals [6]. A similar difference of physiology and pathophysiology of the HPA axis exists between wild-type and *TLR4 *deficient mice. Under physiological conditions the cortex of the adrenal glands is significantly enlarged and plasma concentrations of corticosterone and the pro-inflammatory cytokines tumor necrosis factor (TNF)-α, interleukin (IL)-1β and IL-12 are significantly higher when compared to wild-type animals. Systemic inflammation induces an increase of corticosterone plasma concentration in wild-type, but a decrease in *TLR4 *deficient mice [7].

In humans, single nucleotide polymorphisms (SNPs) are described. For *TLR2 *the most investigated SNP is Arg753Gln which is located in the coding region with a prevalence of approximately 3 to 9.4% in the Caucasian population [8-13]. Children carrying the SNP of Arg753Gln are more susceptible to febrile infections compared to non-carriers [13]. Furthermore, the Arg753Gln polymorphism has been reported to increase the risk of gram-positive and candida sepsis in critical ill patients [8,10], and to increase restenosis rate in patients who underwent percutaneous transluminal coronary angioplasty [14].

The two most investigated SNPs of *TLR4 *are Asp299Gly and Thr399Ile. Six to 14% of the European population are double heterozygote carriers, whereas less than 0.3% carry either the Asp299Gly or the Thr399Ile SNP alone [15]. Compared to non-carriers, Asp299Gly/Thr399Ile carriers demonstrated a blunted decrease of forced expiratory volume in one second in response to LPS inhalation [16,17], and significant lower plasma levels of the inflammatory markers IL-6, IL-1β and C-reactive protein (CRP) in response to LPS injection [18].

Cardiac surgery leads to the activation of both the immune system and the HPA axis. In particular the application of extracorporal circulation, that is, cardiopulmonary bypass (CPB) with distinct contact between blood and artificial surfaces induces complement system, leucocyte activation and the release of cytokines, nitric oxide and oxygen-free radicals [19,20]. The latter pathophysiological changes lead to a systemic inflammatory response and are associated with the release of adrenocorticotropic hormone (ACTH), cortisol [21-23] and various cytokines [24].

In this prospective observational clinical cohort study we aimed to asses the impact of *TLR2 *and *TLR4 *polymorphisms on HPA axis regulation and cytokine release related to systemic inflammation during/following cardiac surgery. Primary endpoint was the influence of *TLR2 *and *TLR4 *SNP on ACTH and cortisol regulation. Secondary endpoint was the influence of *TLR2 *and *TLR4 *SNP on systemic cytokine release.

## Materials and methods

### Patients

This prospective single center observational clinical cohort study was approved by the local ethical review committee (University Hospital Duesseldorf) and carried out in compliance with the principles established in the Helsinki Declaration. Written consent was obtained from 383 patients undergoing elective cardiac surgery (coronary artery bypass graft (CABG) and/or valve surgery (VS) including replacement and reconstruction). Inclusion criteria: age 18 or older, elective cardiac surgery, on CPB. Exclusion criteria: cardiac surgery performed without CPB, history of diseases affecting the HPA axis, systemic or local treatment with glucocorticoids within 30 days before surgery.

### Clinical management

Following standard oral benzodiazepine premedication the night before surgery and one to two hours preoperative on the day of operation, standard monitoring, peripheral venous and arterial access were established prior to induction. Anesthesia was induced with fentanyl (3 to 4 μg/kg) and thiopenthal (1 to 2 mg/kg). Following muscle relaxation with pancuronium bromide (100 μg/kg), the patient was intubated, ventilated and general anesthesia was maintained using fentanyl and sevoflurane (0.8 to 1.5 vol% end-tidal). Central venous access was established, a rectal temperature probe and a urine catheter were inserted. Prior to CPB the patient was fully heparinized with 300 IU/kg heparin i.v. achieving an activated clotting time (ACT) of longer than 400 seconds. Every patient underwent standard nonpulsatile, hypothermic (28°C to 32°C) CPB (roller pump: Stöckert, Munich, Germany; membrane oxygenator: Cobe, Arvada, CO, USA). Flow rate initially started at 2.4 L/minute/m^2 ^and was further adjusted to maintain a mean arterial blood pressure (MAP) of 60 mmHg. Heparin was administered intermittently to maintain ACT between 400 and 500 seconds. Bretschneider solution was used for cardioplegia. At the end of surgery heparin was antagonized with protamine (3 mg/kg) and after re-warming patients' temperature to a minimum of 34°C, CPB was weaned off slowly with fluids and/or inotropic agents infused according to central venous pressure or MAP respectively. Patients, intubated, ventilated and sedated were then transferred to the ICU.

### Sampling

Beside routine pre- and postoperative blood tests three consecutive blood samples were obtained from each patient (supine position). *Sample A (whole blood and serum): *Preoperative, between 07:00 and 09:00; *Sample B (serum): *Postoperative, on arrival to the intensive care unit (ICU); *Sample C (serum): *Postoperative Day 1, between 07:00 and 09:00. Whole blood samples were stored at -80°C, serum samples were centrifuged and stored at -20°C until laboratory analysis.

### DNA preparation and genotyping

DNA was extracted from whole blood by commercial kits (QIAmp, Qiagen, Hilden, Germany). Genotyping for *TLR2 *SNP Arg753Gln (rs5743708) and *TLR4 *SNPs Asp299Gly (rs4986790) and Thr399Ile (rs4986791) was done by melting curve analysis employing FRET probes and the LightcyclerTM (Roche Diagnostics, Mannheim, Germany) as described previously [25]. In brief, 10 to 50 ng genomic DNA was amplified using the following primers: forward: AGTGAGC-GGGATGCCTACT and reverse: GACTTTATCGCAGCTCTCAGATTTAC for *TLR2*; forward: ATTTAAAGAAATTAGGCTTCATAAGCT and reverse: CCAAGAAGTTTG-AACTCATGGTAA for *TLR4*. Hybridisation FRET probes CAAGCTGCAGAAGATAA-TGAACACCAAG-FL and LC Red640-CCTACCTGGAGTGGCCCATGGACG for R753Q gave rise to melting peaks at 60.9°C for the wild-type allele and 65.4°C for the mutated allele. Hybridisation FRET probes CTACTACCTCGATGATATTATTGACTTATT-FL and LC Red640-AATTGTTTGACAAATGTTTCTTCATTTTCC for Asp299Gly and LC Red705-ATTTTGGGACAACCAGCCTAAAGTAT and CTTGAGTTTCAAAGGTTG-CTGTTCTCAAAGT-FL for Thr399Ile gave rise to melting peaks at 62°C and 57.4°C or 67°C and 60.6°C for wild-type and mutated alleles, respectively.

### Measurements of ACTH and cortisol

ACTH and cortisol serum concentrations were measured by radioimmunoassays (Diagnostic System Laboratories Deutschland DSL, Sinsheim, Germany) as recently described [26]. Concentrations are given as pg/ml for ACTH and μg/dl for cortisol.

### Measurements of cytokines

Serum levels of interferon (IFN)-γ, IL-1β, IL-2, IL-4, IL-5, IL-6, IL-8, IL-10, TNF-α and granulocyte macrophage-colony stimulating factor (GM-CSF) (Human Cytokine 10-Plex for Luminex™ laser, BioSource Europe, S.A. Nivelles, Belgium) were determined using the microsphere array technique (Luminex 100 system, Luminex Corp. Austin, TX, USA). Assays were performed according to the manufacturer's protocols [27]. This 10-Plex was chosen because it covers the most important/investigated cytokines in human serum in the context of acute systemic inflammation. Concentrations are given as pg/ml. Detection limits (in pg/ml): IFN-γ: 5, IL-1β: 15, IL-2: 6, IL-4: 5, IL-5: 3, IL-6: 3, IL-8: 3, IL-10: 5, TNF-α: 10, GM-CSF: 15.

### Statistical analysis

Continuous values are displayed as means and 95% confidence intervals or medians with interquartile range. Continuous baseline data were tested for differences between the groups *TLR2 *SNP, *TLR4 *SNP and non-carriers with two-sided Kruskal-Wallis-tests. Categorical values are displayed as frequencies and percentages. Categorical baseline data were tested for differences between groups by two-sided Fisher's exact tests. The time courses of cortisol, ACTH and cytokines were analysed by means of absolute changes from baseline for time points A, B, and C in a linear mixed model. The multiple visits per patient were taken into account. Independence was used as working correlation matrix. Pair wise contrasts were calculated to compare pairs of groups with regard to differences in change from baseline. The factors gender, height, weight, type of surgery, duration of surgery, and outcome of 28-day follow-up were included into the model. Backward selection was used to identify significant factors at a level of 0.05. Also visit and the interaction group visit were included to test for differences in the course of the values over time. Two-sided *P*-values below 0.05 were regarded as statistically significant. Calculations were performed using SAS 9.2 (SAS Institute Inc., Cary, NC, USA).

## Results

### Patient selection, demographic data and baseline characteristics

All patients fulfilling inclusion criteria who granted informed consent were consecutively enrolled over a period of eight months. There were no changes in anesthetic, surgical, or perfusion techniques during this period. A total of 383 patients were included. Patients were excluded who required reoperation within the period of observation (*n *= 12), were unexpectedly operated without CPB (*n *= 7) or received glucocorticoid therapy during or after surgery (*n *= 6). In 10 of the remaining patients genotyping failed for technical reasons. Two more patients identified as SNP carrier for both, *TRL2 *and *TLR4 *were excluded. For the remaining 346 patients, frequency distribution analyzes of cortisol- and ACTH- concentrations in the baseline samples (A) followed. To reduce the undue influence of subjects demonstrating undetected HPA axis pathologies, preoperative systemic inflammation or measurement related discrepancies, outliers were defined as values above 99.5% tolerance intervals (TI) and subjects demonstrating these outliers were excluded from analyzes. A total of 338 patients, all European Caucasians were included; 13 patients were identified as *TLR2*, 51 as *TLR4 *SNP carriers, 274 patients were identified as non-carriers. All *TLR2 *SNP carriers were heterozygous for Arg753Gln, none homozygous. All *TLR4 *SNP carriers were heterozygous for both, Asp299Gly and Thr399Ile. None was heterozygous for Asp299Gly or Thr399Ile only. None was homozygous for Asp299Gly or Thr399Ile, none of the patients was identified to be homozygous for both alleles. Each SNP was in Hardy-Weinberg equilibrium (*TLR2*: *P *= 0.72; *TLR4*: *P *= 0.13). Demographic data, baseline characteristics and 28-day outcome did not differ between non-carriers, *TLR2 *SNP and *TLR4 *SNP carriers with the exception of beta-blocker intake (Table 1). The difference in frequency of beta-blocker intake was further analyzed and found to be absent when comparing non-carriers with *TLR4 *SNP carriers (*P *= 0.1257).

**Table 1:** Demographic data and baseline characteristics of study population.

	Non-carrier	*TLR2 *SNP	*TLR4 *SNP	*P*-value
Number, *n*	274	13	51	
Gender (M/F)	200/74	8/5	38/13	0.6236
Age, yr (median (IQR))	69 (62 to 75)	74 (66 to 77)	71 (61 to 77)	0.1944
Weight, kg (median (IQR))	80 (70 to 90)	80 (70 to 93)	80 (70 to 90)	0.7685
Height, m (median (IQR))	1.72 (1.65 to 1.76)	1.68 (1.65 to 1.72)	1.73 (1.66 to 1.78)	0.2200
Time of surgery, h	4.0 (3.4 to 4.7)	3.8 (3.3 to 4.1)	3.8 (3.3 to 4.4)	0.2101
Diabetes mellitus, *n *(%)	96 (35)	5 (38)	12 (24)	0.2375
Admission medication				
Beta-blocker, *n *(%)	215 (78)	13 (100)	35 (69)	0.0362
ACE inhibitor, *n *(%)	137 (50)	10 (77)	30 (59)	0.1048
Calcium channel-blocker, *n *(%)	39 (14)	4 (31)	4 (8)	0.0862
Diuretics, *n *(%)	138 (50)	5 (38)	25 (49)	0.7455
Nitrates, *n *(%)	68 (25)	3 (23)	11 (22)	0.9285
Type of surgery				
CABG, *n *(%)	183 (67)	9 (69)	36 (71)	
VS, *n *(%)	33 (12)	3 (23)	4 (8)	0.7542
CABG + VS, *n *(%)	43 (16)	1 (8)	7 (14)	
Other, *n *(%)	15 (6)	0 (0)	4 (8)	
28-day outcome				
Survivor, *n *(%)	247 (93)	9 (75)	41 (89)	0.0753
Nonsurvivor, *n *(%)	19 (7)	3 (25)	5(11)	

CABG, coronary artery bypass graft; IQR, interquartile range; *TLR2 *SNP, toll-like receptor2 single-nucleotide polymorphism carrier; *TLR4 *SNP, toll-like receptor4 single-nucleotide polymorphism carrier; VS, valve surgery.

### ACTH and cortisol

**Figure 1:**
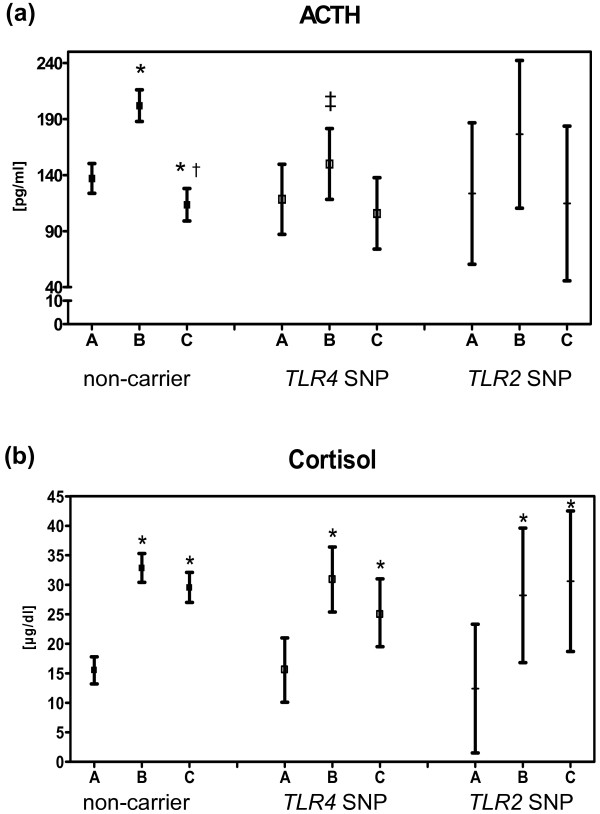
***TLR4*/*TLR2 *polymorphisms and time course of perioperative serum concentrations of adrenocorticotropic hormone (ACTH) (a) and cortisol (b)**. Sampling times: A: preoperative, B: postoperative at day of surgery, C: postoperative Day 1. *TLR2 *SNP, toll-like receptor2 single-nucleotide polymorphism carrier; *TLR4 *SNP, toll-like receptor4 single-nucleotide polymorphism carrier. Number of patients in each group: non-carrier = 274, TLR4 SNP = 51, TLR2 SNP = 13. Values are shown as mean and 95% confidence intervals. *P < 0.05 compared to *A*; †*P *< 0.05 compared to *B*; ‡*P *< 0.05 compared to non-carrier.

Basal ACTH and cortisol serum levels did not differ between the three genotypes (Figure 1a, b). In all three genotypes cortisol levels significantly raised postoperatively at sample times B and C. However, only in the non-carrier group this was accompanied by a significant ACTH rise. At sample time C there was a significant decrease of the ACTH levels compared to sample points A and B in the non-carrier group. Neither in the *TLR4 *SNP nor in the *TLR2 *SNP carrier group there was a significant difference in changes of ACTH serum concentrations. At sample time B the absolute changes of ACTH levels were significantly different between non-carriers and *TLR4 *SNP carriers.

### Cytokines

**Figure 2:**
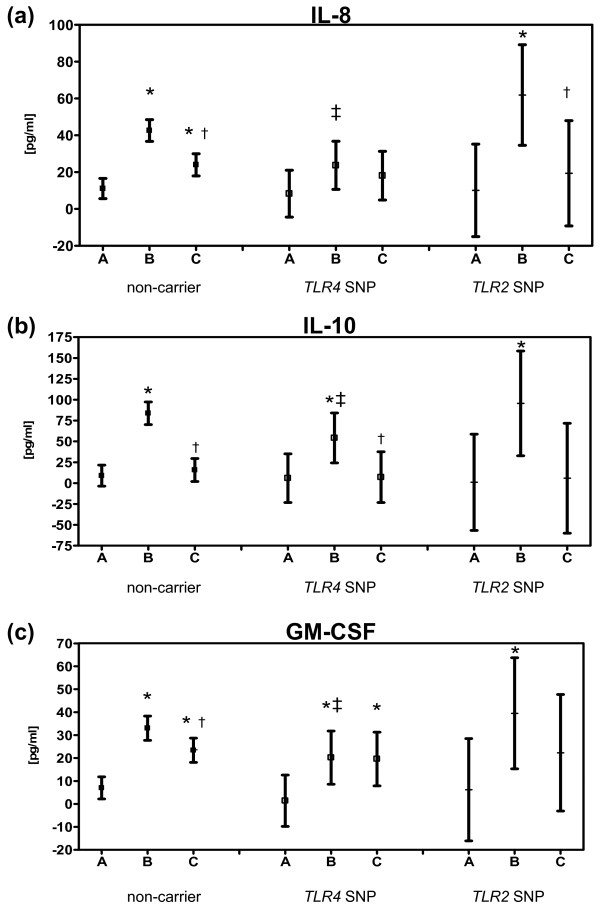
***TLR4*/*TLR2 *polymorphisms and time course of perioperative serum concentrations of IL-8 (a), IL-10 (b) and GM-CSF (c)**. Sampling times: A: preoperative, B: postoperative at day of surgery, C: postoperative Day 1. GM-CSF, granulocyte macrophage-colony stimulating factor; IL, interleukin; *TLR2 *SNP, toll-like receptor 2 single-nucleotide polymorphism carrier; *TLR4 *SNP, toll-like receptor 4 single-nucleotide polymorphism carrier. Number of patients in each group: non-carrier = 274, TLR4 SNP = 51, TLR2 SNP = 13. Values are shown as mean and 95% confidence intervals. *P < 0.05 compared to *A*; †*P *< 0.05 compared to *B*; ‡*P *< 0.05 compared to non-carrier.

Basal cytokine levels did not differ between the three genotypes (Figure 2a-c, Table 2). Levels of IFN-γ in the majority of the measurements were below the detection limit and therefore not analyzed. No significant changes over time or between the groups were found for the cytokines IL-1β, IL-2, IL-4, IL-5 and TNF-α (Table 2). IL-6 levels significantly rose on sample time B for all genotype groups and on sample time C for non-carriers and *TLR4 *SNP carriers. There was a significant decline in the non-carrier group from sample time B to C. No significant differences were found between the genotype groups (Table 2). IL-8 levels were significantly elevated on sample times B and C, declining significantly from B to C in the non-carrier group. In the *TLR4 *SNP carrier group no significant rise, but even significant lower IL-8 concentrations compared to non-carriers, could be observed. However, there was a transient, significant peak of IL-8 levels in the *TLR2 *SNP group, represented by a significant rise from A to B and a significant drop from B to C (Figure 2a). IL-10 levels peaked at sample time B, that is, significantly increased from A to B and subsequently significantly dropped from B to C in the non-carrier and *TLR4 *SNP group. IL-10 peak concentrations were significantly lower in *TLR4 *SNP carriers compared to non-carriers (Figure 2b). GM-CSF levels rose significantly from A to B in all three groups and from A to C in non-carrier and *TLR4 *SNP carrier patients. In the non-carrier group GM-CSF levels dropped significantly from B to C. GM-CSF levels at sample time B were significantly lower in the *TLR4 *SNP group compared to non-carriers (Figure 2c).

**Table 2:** *TLR4*/*TLR2 *polymorphisms and time course of perioperative cytomine serum concentrations

		Sample times		
		
		A	B	C
**IL-1β **(pg/ml)	non-carrier	8.2 (3.5 to 12.8)	14.4 (9.3 to 19.4)	9.9 (4.8 to 15.0)
	*TLR4 *SNP	6.7 (-4.1 to 17.5)	12.3 (1.2 to 23.5)	11.5 (0.3 to 22.7)
	*TLR2 *SNP	1.2 (-20.2 to 22.6)	29.3 (6.0 to 52.6)	12.4 (-12.0 to 36.9)
**IL-2 **(pg/ml)	non-carrier	2.6 (1.3 to 3.9)	3.0 (1.7 to 4.4)	2.4 (1.0 to 3.8)
	*TLR4 *SNP	2.0 (-1.0 to 5.0)	2.8 (-0.2 to 5.9)	3.3 (0.2 to 6.4)
	*TLR2 *SNP	1.1 (-4.8 to 7.0)	1.8 (-4.6 to 8.2)	1.0 (-5.7 to 7.8)
**IL-4 **(pg/ml)	non-carrier	4.9 (2.7 to 7.1)	4.6 (2.2 to 7.0)	4.2 (1.8 to 6.6)
	*TLR4 *SNP	1.7 (-3.5 to 6.8)	2.0 (-3.3 to 7.3)	1.8 (-3.6 to 7.2)
	*TLR2 *SNP	4.5 (-5.7 to 14.7)	2.4 (-8.7 to 13.5)	3.1 (-8.6 to 14.7)
**IL-5 **(pg/ml)	non-carrier	1.5 (0.7 to 2.4)	2.6 (1.7 to 3.6)	1.5 (0.6 to 2.5)
	*TLR4 *SNP	0.9 (-1.1 to 2.9)	1.4 (-0.7 to 3.5)	0.7 (-1.4 to 2.9)
	*TLR2 *SNP	1.1 (-3.0 to 5.1)	1.4 (-3.0 to 5.8)	0.8 (-3.8 to 5.36)
**IL-6 **(pg/ml)	non-carrier	18.8 (-48.4 to 86.0)	554.1 (481.7 to 626.4)^*a*^	350.4 (277.1 to 423.7)^*ab*^
	*TLR4 *SNP	24.0 (-131.6 to 179.7)	422.5 (262.1 to 583.0)^*a*^	344.7 (182.6 to 506.9)^*a*^
	*TLR2 *SNP	14.3 (-294.0 to 322.7)	696.3 (361.1 to 1031.5)^*a*^	405.7 (54.1 to 757.2)
**TNF-α **(pg/ml)	non-carrier	4.9 (-7.6 to 17.4)	12.9 (-0.6 to 26.3)	8.7 (-4.9 to 22.3)
	*TLR4 *SNP	0.3 (-28.5 to 29.2)	1.9 (-27.8 to 31.7)	1.0 (-29.1 to 31.1)
	*TLR2 *SNP	0.3 (-56.9 to 57.5)	3.4 (-58.8 to 65.5)	1.5 (-63.7 to 66.7)

Sampling times: A: preoperative, B: postoperative at day of surgery, C: postoperative Day 1. IL, interleukin; *TLR2 *SNP, toll-like receptor2 single-nucleotide polymorphism carrier; *TLR4 *SNP, toll-like receptor4 single-nucleotide polymorphism carrier; TNF, tumor necrosis factor. ^*a*^*P *< 0.05 compared to A; ^*b*^*P *< 0.05 compared to B. Data are given as mean and 95% confidence intervals.

## Discussion

Systemic inflammation, as a result of major surgery or sepsis, has a distinct effect on the immune-adrenal crosstalk. We report for the first time of an association between the presence of a SNP (here: *TLR4*) and perioperative ACTH levels. Changes of ACTH levels were significantly lower in the *TLR4 *SNP carrier group compared to non-carriers. Both, *TLR4 *SNP carriers and non-carriers showed a significant rise of cortisol serum levels following cardiac surgery. This rise was preceded/accompanied by a significant ACTH rise only in non-carriers. Furthermore, our results link for the first time a SNP (here: *TLR4*) with differences in perioperative time courses of IL-8, IL-10 and GM-CSF serum levels, that is, in contrast to non-carriers, *TLR4 *SNP carriers demonstrated significantly lower immediate postoperative serum concentrations.

Major surgery, for example, cardiac surgery with CPB, leads to a systemic inflammation which is accompanied by an activation of the HPA axis [28,29]. A significant rise of postoperative serum cortisol in cardiac surgery patients has been described in several studies over the last decades [30-34]. The rise of endocrine stress markers seems not to depend on the individual, anticipatory stress of the patient awaiting surgery, the type of postoperative respiratory weaning, perioperative beta blockade or sufentanil or fentanyl doses [35-39].

Dissociation between cortisol and ACTH levels following major surgery has been observed, particularly on the first postoperative day, whereas ACTH levels spread strongly immediately after surgery [23,37,40,41]. ACTH is produced primarily by the anterior pituitary gland. Alternative sources described in the literature are immunocompetent cells, adrenal gland and inflammatory sites [42-45]. Furthermore, there are hints, that the splanchnic nerve is involved in adrenal cortex regulation [46,47]. As we observed a similar release of cortisol in *TLR4 *SNP carriers and non-carriers, the above mentioned alternative adrenal cortex stimuli can be discussed as compensatory mechanisms for cortisol release in *TLR4 *SNP carriers. Therefore, one could speculate that in *TLR4 *SNP carriers, cortisol release might be rather locally triggered, while adrenal glands of non-carriers are mainly controlled by systemic ACTH. In an ACTH stimulation study in 45 cardiac surgery patients, 11 (25%) had an impaired cortisol response [48]. These effects could be explained by our findings in that *TLR4 *SNP carriers were part of the patient population.

Pro-inflammatory cytokines are involved in the release of corticotropin releasing hormone (CRH) and subsequent ACTH release [49]. In CRH-knockout mice viral infection leads to an ACTH independent corticosterone response, which is associated with significantly higher IL-6 plasma concentrations compared to WT mice [50]. This could be interpreted as exaggerated IL-6 levels compensating for the lack of ACTH. However, in our study changes in IL-6 levels did not differ between the groups. Also, GM-CSF is able to trigger cortisol release [51]; however, we found changes in GM-CSF concentration to be significantly *lower *in the *TLR4 *SNP carrier group. None of the measured cytokines were found to be higher up-regulated in the *TLR4 *SNP group compared to the non-carrier cohort. Therefore, our data do not support the concept of cytokines being compensatory up-regulated counterbalancing low ACTH levels to allow sufficient cortisol levels.

Also, in critically ill patients dissociations between ACTH and cortisol have been described, particularly from days 4 to 5 post trauma or beginning of sepsis [52]. A clinical study (Corticus) including patients with severe sepsis or septic shock demonstrated that survivors had lower baseline cortisol levels and significant higher Δmax (that is, peak cortisol following ACTH stimulation minus baseline cortisol) compared to nonsurvivors [53]. In critically ill, for example, septic patients, adrenal insufficiency can occur and it has been postulated that particularly these patients could benefit from a therapy with glucosteroids [54,55]. However, the diagnosis of adrenal insufficiency in critically ill is difficult and there is still an ongoing search for an adequate diagnostic tool. The diagnosis of adrenal insufficiency in septic patients examined in multicenter trials is complicated by a high inter-assay variation [56]. In a recently published recommendation upon the diagnosis and management of corticosteroid insufficiency in critically ill adult patients, the standard ACTH stimulation test for diagnosing adrenal insufficiency is not recommended to be performed as a routine [57]. In literature, the prevalence of adrenal insufficiency in critically ill patients varies widely between the studies (0 to 77%) [52,57,58]. The question arising from our results is: How do adrenal glands from *TLR4 *SNP carriers respond to an ACTH stimulation test as they are obviously releasing cortisol less dependent/independent from ACTH during systemic inflammation? Does ACTH stimulation result in even higher cortisol serum levels or are adrenal glands of *TLR4 *SNP carriers insensitive to ACTH? This should be taken into consideration when interpreting completed, and planning for new clinical trials on HPA axis regulation in septic patients. Particularly, considering the fact that *TLR4 *SNP carriers demonstrate a higher risk for developing sepsis, that is, the frequency of *TLR4 *SNP carriers in a septic patient cohort is higher (approximately 20%) compared to the normal population or, for example, cardiac surgical patients [59-61].

A perioperative rise of several cytokines following major/cardiac surgery is well described [19,28,29,62]. Regarding perioperative TNF-α and IL-6 plasma concentrations and the influence of *TLR4 *SNP (Asp299Gly/Thr399Ile), our findings are similar to previous trials. In abdominal surgical patients neither non-carriers nor *TLR4 *SNP carriers showed a significant rise in TNF-α postoperatively. However, both cohorts demonstrated a significant IL-6 rise compared to preoperative baseline concentrations. Neither TNF-α, nor IL-6 plasma concentrations differed significantly between non-carriers and *TLR4 *SNP carriers [63]. Furthermore, there was no difference of IL-6 levels in a healthy population (8 *TLR4 *SNP *vs *49 non-carriers) treated with low dose LPS [64]. A diminished Human Leukocyte Antigen (HLA-DR) expression on monocytes and B-lymphocytes following cardiac surgery can *in vitro *be reversed by GM-CSF [65]. One could therefore speculate, that *TLR4 *SNP carriers would express less HLA-DR, possibly making them more susceptible to postoperative infections. On the other hand IL-10 can reverse HLA-DR up-regulation [66]. Translated to our results this would mean better immune competence of *TLR4 *SNP carriers. Post surgery and/or trauma IL-8 and IL-10 plasma levels are significantly higher in nonsurvivors compared to survivors [67], which would translated to our study result in a higher mortality in the non-carrier group. However, our study did not find differences in 28-day outcome between the cohorts, which might be due to an underpowered sample size.

*Ex vivo *stimulation of whole blood or isolated monocytes revealed similar cytokine responses as observed in our study: The presence of *TLR4 *SNP did not influence the LPS induced release of TNF-α, IL-1β or IL-6 compared to non-carriers. However, comparable to our results, *TLR4 *SNP led to a reduced IL-10 release [68,69]. With IL-10 enhancing ACTH release [70], the significant lower rise of IL-10 in *TLR4 *SNP carriers could have accounted for the absence of a significant change in ACTH levels in this genotype.

During cardiac surgery, phases of hypoperfusion with consecutive tissue hypoxia occur. Hypoxia induces expression and increases signaling of TLRs [71]. This seems to be particularly true for TLR2 and TLR6 [72]. You could therefore speculate that hypoxia induced TLR2 expression is negatively influenced by the presence of *TLR2 *SNP. The *TLR2 *SNP carrier group in this study is probably too small to estimate whether the SNP for *TLR2 *would have an effect on, for example, cytokine release. Serum concentrations of cytokines investigated in this study do not differ between non-carriers and *TLR2 *SNP carriers.

A study in patients (*n *= 94) being admitted to the intensive care unit for various reasons (sepsis, cardiovascular failure, pancreatitis, respiratory failures, and so on) failed to demonstrate a correlation between SNP *TLR4 *Asp299Gly and length of stay (hospital or intensive care). However, mortality was higher in *TLR4 *SNP carriers [61]. Our study did not find a correlation between hospital or intensive care length of stay (data not shown) or mortality (see results) and *TLR2 *or *TLR4 *SNP. As a further limitation of this study, the *TLR2 *SNP carrier group compared to the non-carrier group is too small to draw major conclusions. Also, the study is underpowered for detecting significant differences in morbidity or mortality between the cohorts. Further studies are needed to determine if the observations made in this study have any impact on clinical outcome.

With this study we translated observations made in animals to a clinical scenario. In *TLR2 *and *TLR4 *knockout mice we demonstrated the altered regulation of HPA axis and cytokines during systemic inflammation. In patients, polymorphisms of *TLR2 *and *TLR4 *influence HPA axis and cytokine response to surgical stress, that is, systemic inflammation.

## Conclusions

In conclusion, this clinical study in cardiac surgical patients demonstrates a diminished perioperative ACTH release in *TLR4 *SNP carrying patients. Carriers and non-carriers, however, demonstrated the same transient, perioperative rise in cortisol serum concentrations, indicating that in *TLR4 *polymorphism carriers, cortisol release seems to be less dependent or even independent of systemic ACTH concentrations. These findings should be considered when diagnosing and treating adrenal insufficiency in patients with systemic inflammation, for example, sepsis. Furthermore, *TLR4 *SNP carriers demonstrated a significantly reduced release of the cytokines IL-8, IL-10 and GM-CSF compared to non-carriers. TNF-α, IL-1β, IL-2, IL-4, IL-5 and IL-6 did not differ between *TLR4 *SNP carriers and non-carriers.

## Key messages

• Cardiac surgical patients carrying a genetic variation of *TLR4 *demonstrate diminished perioperative ACTH release.

• However, postoperative cortisol rise did not differ from non-carriers, indicating ACTH not to be the primary stimulus for perioperative cortisol release in *TLR4 *carriers.

• This finding might have impact on interpreting previous and planning new studies investigating adrenal insufficiency in patients with systemic inflammation (for example, sepsis).

• *TLR4 *polymorphism carriers demonstrated lower postoperative peaks of the cytokines IL-8, IL-10 and GM-CSF.

## Abbreviations

ACT: activated clotting time; ACTH: adrenocorticotropic hormone; CABG: coronary artery bypass graft; CPB: cardiopulmonary bypass; CRH: corticotropin releasing hormone; CRP: C-reactive protein; DNA: deoxyribonucleic acid; GM-CSF: granulocyte macrophage-colony stimulating factor; HLA-DR: Human Leukocyte Antigen; HPA: hypothalamic-pituitary-adrenal; ICU: intensive care unit; IFN: interferon; IL: interleukin; LPS: lipopolysaccharide; LTA: lipoteichoic acid; MAP: mean arterial blood pressure; SNP: single nucleotide polymorphisms; TLR: Toll-like receptor; TNF: tumor necrosis factor; VS: valve surgery.

## Competing interests

The authors declare that they have no competing interests.

## Authors' contributions

AK contributed to idea and design of the study, was responsible for acquisition of patient data, collected and analyzed the data and wrote the manuscript. LH and RRS performed SNP analyzes and contributed to the drafts of the manuscript. MS performed ACTH and cortisol analyzes and contributed to the drafts of the manuscript. OB and contributed to the writing of the paper, collected data and assisted in patient recruitment. DG and MK collected data and assisted in patient recruitment. SRB helped to design the study and participated in the interpretation of all data. CS performed statistical analyzes. KZ conceived of the study, obtained funding, participated in its design and coordination, headed the project and helped to draft the manuscript. All authors read and approved the final manuscript.
